# Does PEEK/HA Enhance Bone Formation Compared With PEEK in a Sheep Cervical Fusion Model?

**DOI:** 10.1007/s11999-016-4994-x

**Published:** 2016-08-22

**Authors:** William R. Walsh, Matthew H. Pelletier, Nicky Bertollo, Chris Christou, Chris Tan

**Affiliations:** Surgical & Orthopaedic Research Laboratories, Prince of Wales Clinical School, Prince of Wales Hospital, UNSW Australia, Level 1 Clinical Sciences Building, Randwick, NSW 2031 Australia

## Abstract

**Background:**

Polyetheretherketone (PEEK) has a wide range of clinical applications but does not directly bond to bone. Bulk incorporation of osteoconductive materials including hydroxyapatite (HA) into the PEEK matrix is a potential solution to address the formation of a fibrous tissue layer between PEEK and bone and has not been tested.

**Questions/purposes:**

Using in vivo ovine animal models, we asked: (1) Does PEEK-HA improve cortical and cancellous bone ongrowth compared with PEEK? (2) Does PEEK-HA improve bone ongrowth and fusion outcome in a more challenging functional ovine cervical fusion model?

**Methods:**

The in vivo responses of PEEK-HA Enhanced and PEEK-OPTIMA^®^ Natural were evaluated for bone ongrowth in the form of dowels implanted in the cancellous and cortical bone of adult sheep and examined at 4 and 12 weeks as well as interbody cervical fusion at 6, 12, and 26 weeks. The bone-implant interface was evaluated with radiographic and histologic endpoints for a qualitative assessment of direct bone contact of an intervening fibrous tissue later. Gamma-irradiated cortical allograft cages were evaluated as well.

**Results:**

Incorporating HA into the PEEK matrix resulted in more direct bone apposition as opposed to the fibrous tissue interface with PEEK alone in the bone ongrowth as well as interbody cervical fusions. No adverse reactions were found at the implant–bone interface for either material. Radiography and histology revealed resorption and fracture of the allograft devices in vivo.

**Conclusions:**

Incorporating HA into PEEK provides a more favorable environment than PEEK alone for bone ongrowth. Cervical fusion was improved with PEEK-HA compared with PEEK alone as well as allograft bone interbody devices.

**Clinical Relevance:**

Improving the bone–implant interface with a PEEK device by incorporating HA may improve interbody fusion results and requires further clinical studies.

## Introduction

Interbody spinal fusion has a long history from the pioneering works of Cloward [10] and Babgy [1]. The biomechanical benefits of interbody fusion and spinal instrumentation in the treatment of disorders of the spine are firmly established [11]. The evolution of interbody devices has dramatically changed from a design standpoint as well as the materials they are made from as is evident in the literature [7, 22].

Polyetheretherketone (PEEK) is a biocompatible semicrystalline thermoplastic polymer that has a wide range of clinical applications [29]. PEEK has been used in spinal fusion surgeries, predominantly in the form of load-bearing interbody cages, for nearly 15 years [16]. Clinical studies continue to support that PEEK performs as well as, or better than, equivalent interbody fusion devices made of metals or allograft while providing some distinct clinical and manufacturing advantages [6, 8, 17]. These advantages include mechanical strength, a modulus similar to cortical bone, imaging compatibility, biocompatibility, and ease of manufacture. Clinically, interbody cages allow graft material to be contained within the device and participate in the fusion, be it autograft, allograft, or synthetic bone graft substitutes [18]. Nevertheless, bone does not directly bond to PEEK [20–22], which is a reflection of the chemical inertness and hydrophobic nature of the material [19]. In vivo, a nonreactive, discontinuous fibrous tissue interface can form that has been well reported in preclinical models [28, 30] and clinically observed based on imaging modalities [21]. High fusion rates and low subsidence rates, however, are consistently reported for PEEK-based devices [7].

Bulk incorporation of osteoconductive materials including hydroxyapatite (HA) into the PEEK matrix is one potential solution to address the fibrous tissue layer present between PEEK and bone [21]. Materials science research into the combination of PEEK and HA has evaluated processing as well as bioactivity of this composite material [12, 27, 31].

We hypothesized that the incorporation of HA into the PEEK matrix would enhance in vivo response at the bone–implant interface. Using in vivo ovine animal models, we asked: (1) Does PEEK-HA improve cortical and cancellous bone ongrowth compared with PEEK? (2) Does PEEK-HA improve bone ongrowth and fusion outcome in a more challenging functional ovine cervical fusion model? The null hypothesis was that there were no differences between the different materials at the bone–implant interface.

## Materials and Methods

Local institutional animal ethical approval was obtained before the start of this work. The local histologic reaction at the bone–implant interface was evaluated in a well-reported and standardized bone–implant interface model in both cortical and cancellous bone in sheep [2–5, 24–26, 28] in the form of cylindrical dowels. Unfilled PEEK-OPTIMA^®^ Natural (PEEK; Invibio Limited, Hillhouse International, Thornton-Cleveleys, Thornton, Cleveleys, UK) served as the predicate material, whereas PEEK-OPTIMA^®^ HA Enhanced (PEEK-HA) was the test material. The HA in the PEEK-OPTIMA^®^ HA Enhanced has been fully dispersed in the PEEK matrix with mechanical properties similar to the standard unfilled PEEK-OPTIMA^®^ Natural.

Dowels (6 mm diameter × 25 mm long) were prepared from bar stock of PEEK and PEEK-HA and autoclaved before surgery. Three bicortical defects (6 mm diameter) were created with a three-fluted pyramidal tip 4.5-mm drill (Surgibit; Orthopedic Innovations, Collaroy, Australia) overdrilled with a 6-mm drill. Dowels were implanted in the cortical bone of the tibia in a line-to-line fashion with a spacing of 20 mm. Cancellous implants were inserted in a press-fit manner after the creation of 5.5-mm defects in the cancellous bone of the proximal tibia and distal femur. The sample size in the cortical sites was n = 5 for both groups, whereas the sample size in the cancellous bone was n = 4 for PEEK and n = 3 for PEEK-HA at 4 and 12 weeks. One dowel of each group was examined before surgery using a stereo-zoom microscope and an environmental scanning electron microscope to assess the surface when HA was incorporated into PEEK.

The surgical sites were inspected for any signs of adverse reactions to the implants in terms of infection, inflammation, or swelling. The femur and tibias were radiographed in the craniocaudal and lateral planes using a Faxitron machine (Faxitron X-ray Corporation, Tucson, AZ, USA) and digital plates. The DICOM data were examined using an ezDICOM medical viewer (www.mricro.com) to evaluate the implant-bone interface from a radiographic perspective for evidence of adverse events at the implant-bone interface in terms of bony resorption.

The cortical and cancellous samples were processed for hard tissue histology by fixation in 10% phosphate-buffered formalin, ethanol dehydration (70%–100%), infiltration with methylmethacrylate (MMA), and polymerization in polymethylmethacrylate (PMMA). PMMA-embedded cortical implant dowels were sectioned along the long axis of the implants using a Leica SP 1600 Microtome (Leica, Melbourne, Australia). The medial and lateral cortical specimens were sectioned in the middle of the implant. Two sections were cut from each PMMA block (approximately 15–20 μm) and stained with methylene blue and basic fuchsin. PMMA-embedded cancellous implant dowels were sectioned perpendicular to the long axis of the implant (approximately 15–20 μm) and were cut and stained at 5-mm increments for the cancellous implantations. The bone–implant interfaces on the superior and inferior aspects of the implant were examined using × 2 objective using an Olympus microscope and a DP72 video camera (Olympus, Tokyo, Japan) to obtain a digital image of the entire interface to evaluate bone or fibrous tissue ongrowth. The implant-bone interface was examined at higher magnification for general tissue response, and the presence of inflammatory cells as outlined in ISO 10993-6 Biological evaluation of medical devices–Part 6: Test of local effects after implantation [23]. The histologic evaluation considered cell type, neovascularization, and fibrous tissue response with a qualitative scale from 0 to 4 [23].

### Cervical Interbody Fusion Study

The in vivo response at the bone-implant interface was further evaluated in a functional interbody cervical fusion model in adult sheep [13–15, 30] using local autograft as the graft material to fill the interbody cages. PEEK and gamma-irradiated ovine allograft cages served as the predicate cage device, whereas cages made from PEEK-HA were the test devices.

Cervical interbody fusion devices of identical design were machined from PEEK, PEEK-HA, or ovine allograft bone. The cage dimensions were 7 × 11 × 14 mm based on anatomic dimensions of the adult ovine cervical spine obtained from CT scans at the study site of adult sheep. The allograft cages were machined from the metatarsus of 2-year-old ovine donors from other studies. The allograft cages were sonicated in 70% ethanol for 30 minutes before air-drying in a laminar flow cabinet. Samples were gamma radiation-sterilized at 25 kGy on dry ice and then stored frozen at −20°C until implantation. All allograft cages were inspected before implantation using Faxitron radiographs and visual inspection to verify they were free from defects at the time of implantation.

The cervical fusion model incorporated 18 fully mature female sheep (4–5 years old) that were randomly assigned to three groups to undergo cervical fusion at two nonadjacent spinal levels (C2–C3 and C4–C5). Preoperative preparation began with fentanyl patches (100 mg at 2 μg/kg/hr transdermally) applied to the sheep 24 hours before surgery for analgesia [9].

With the animal placed in dorsal recumbency, a single ventral midline skin incision was made from the level of the larynx to the manubrium of the sternum. The C2–3 and C4–5 levels were identified through manual of the atlas (C1) after surgical exposure. The ventral aspect of the annulus fibrosis was excised with a No. 10 scalpel blade and the nucleus pulposus was removed with a curette to the level of the endplate. The endplates were prepared with a series of rasps to remove the disc material with preservation of subchondral bone. The interbody cages were filled with local bone harvested from the ventral aspects of the vertebral bodies with a rongeur. A 28-mm four-hole titanium alloy cervical plate with four screws was positioned ventrally to span the treated level. All interbody devices were successfully implanted with no evidence of fracture or damage. The fascia and skin were reapproximated and closed using 2-0 Vicryl suture. The animals were returned to their pens and allowed unrestricted movement after surgery. All animals recovered well after surgery with no adverse events. Animals were euthanized at 6, 12, and 26 weeks as per the study design for radiographic and histologic endpoints.

The cervical spine from C1 to C6 was harvested at the designated time points. High-definition radiographs were using a Faxitron machine (Faxitron X-ray Corporation) and digital plates. The DICOM data from the Faxitron radiographs were examined using ezDICOM medical viewer to evaluate the implant-bone interfaces from a radiographic perspective for evidence of adverse events in terms of bony resorption. Micro-CT was performed using an Inveon Scanner (Siemens, Malvern, PA, USA). Slice thickness was set to approximately 50 μm. Fusions were graded based on the amount and quality of the bone within the interbody devices as well as the contact with the device itself in the sagittal and coronal planes in a blinded fashion (WRW, CT, CC) based on a semiquantitative scale (Table 1).

**Table 1:** CT grading scale

New bone formation in the fusion as well as the device surfaces
0 – None detected
1 – Small uncommon foci
2 – Moderate-sized, multiple foci
3 – Extensive, multiple, coalescing foci
Quality of new bone formation bridging in the fusion as well as the device surfaces
0 – No bridging by new bone
1 – Minor bridging in < 30% of the interface
2 – Partial bridging in 30%–70% of the interface
3 – Extensive bridging in 70+ of the interface
Direct new bone–device contact
0 – No contact by new bone
1 – Minor contact in < 30% of the interface
2 – Partial contact in 30%–70% of the interface
3 – Extensive contact in 70+ of the interface

The fusions were fixed in phosphate-buffered formalin, dehydrated through increasing concentrations of ethanol (70%–100%), infiltrated with MMA, and polymerized in PMMA. Three sections were taken in the sagittal plane with a Leica SP 1600 microtome. Samples were stained using methylene blue (Sigma-Aldrich, Castle Hill, NSW Australia; 1% in borax buffer [0.1 M], pH 8.5) for 1 minute followed by basic fuchsin (Sigma-Aldrich; 0.3% in water). The histology was examined blinded (WRW, PC) to treatment and time for device integrity, endplate interactions, new bone and quality within the interbody device, and local cellular reactions at the bone–implant interface following the guidelines for histology grading scale as outlined in ISO 10993-6 Biological evaluation of medical devices–Part 6: Test of local effects after implantation [23].

## Results

### Adult Ovine Unloaded Bone–Implant Interface

**Fig. 1A–B:**
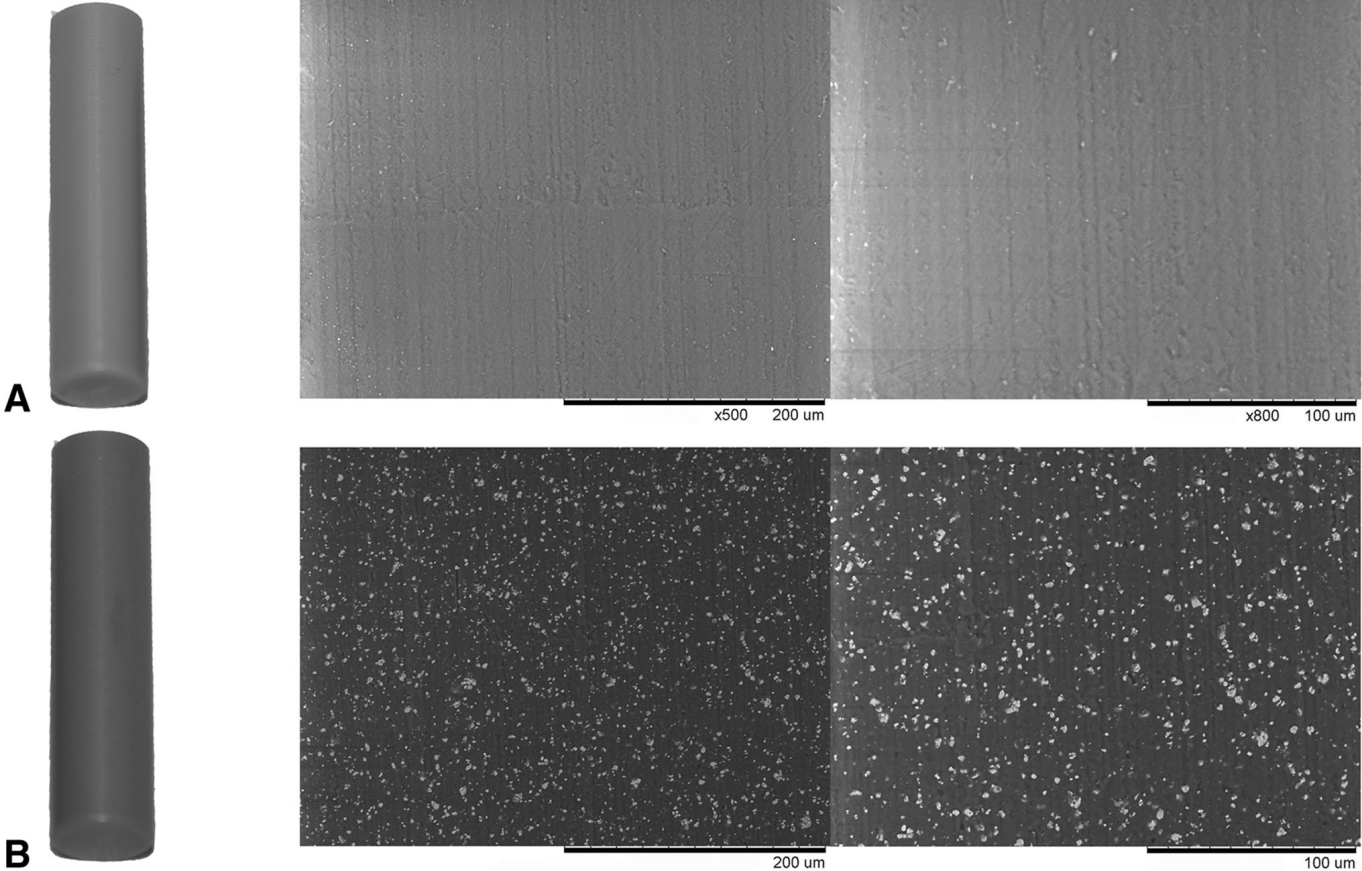
Macroscopically, PEEK-HA (**B**) appears slightly gray compared with PEEK (**A**). Backscattering scanning electron microscopy at ×100 and ×500 demonstrates comparable surface topography and the presence of the HA incorporated into PEEK-HA that is present on the surface of the material and appears as white particulate under electron microscopy.

PEEK-HA implants had similar surfaces compared with PEEK although they appeared slightly grayer as a result of the incorporation of the HA, which was present at the surface (Fig. 1).

**Fig. 2A–D:**
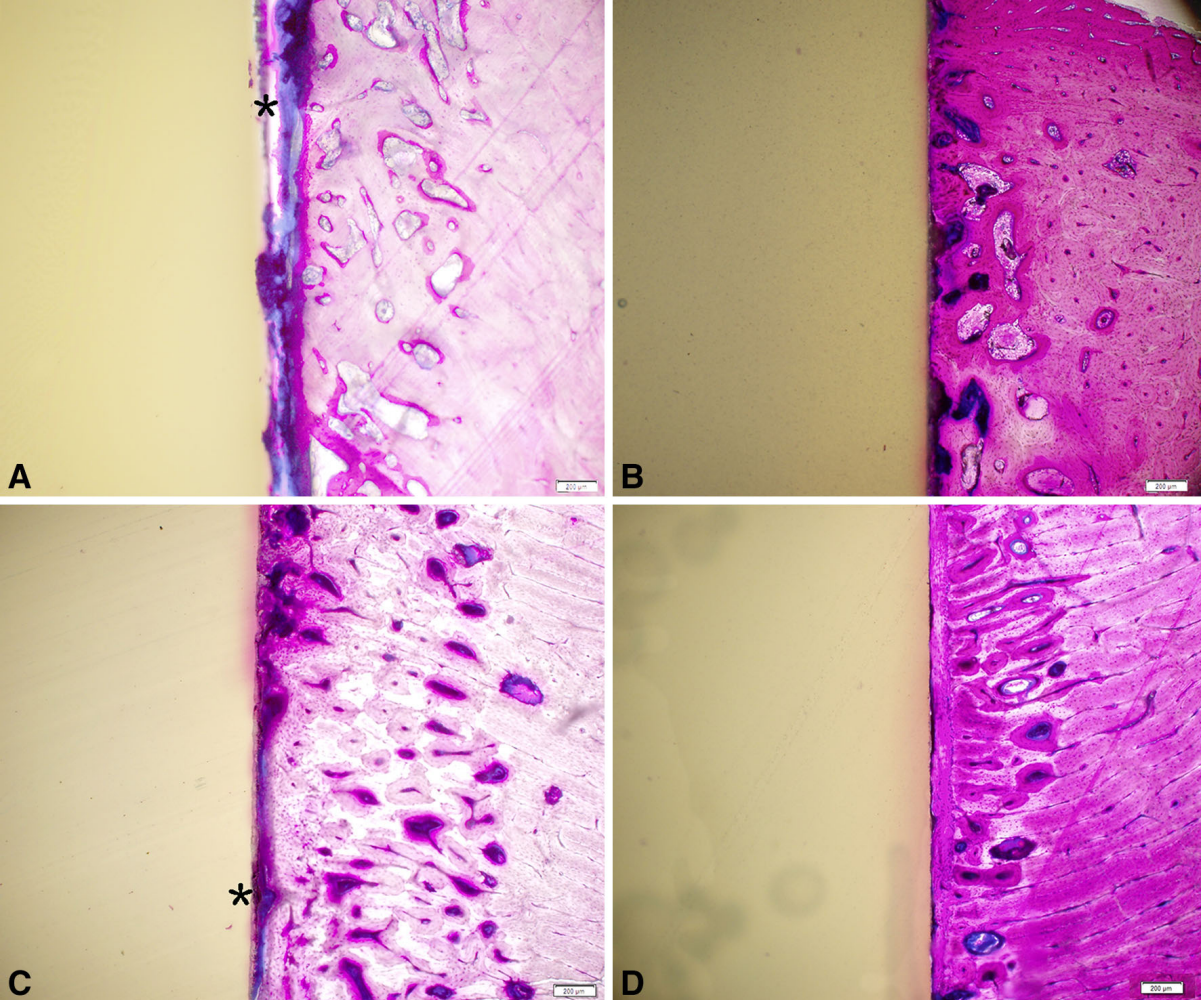
Bone ongrowth in cortical sites for PEEK at 4 and 12 weeks (**A**, **C**) and PEEK-HA (**B**, **D**) demonstrated the presence of fibrous tissue interface for PEEK (*), whereas a direct bone-to-implant interface was observed for PEEK-HA at the magnification used. Magnification bar = 200 μm.

**Fig. 3A–D:**
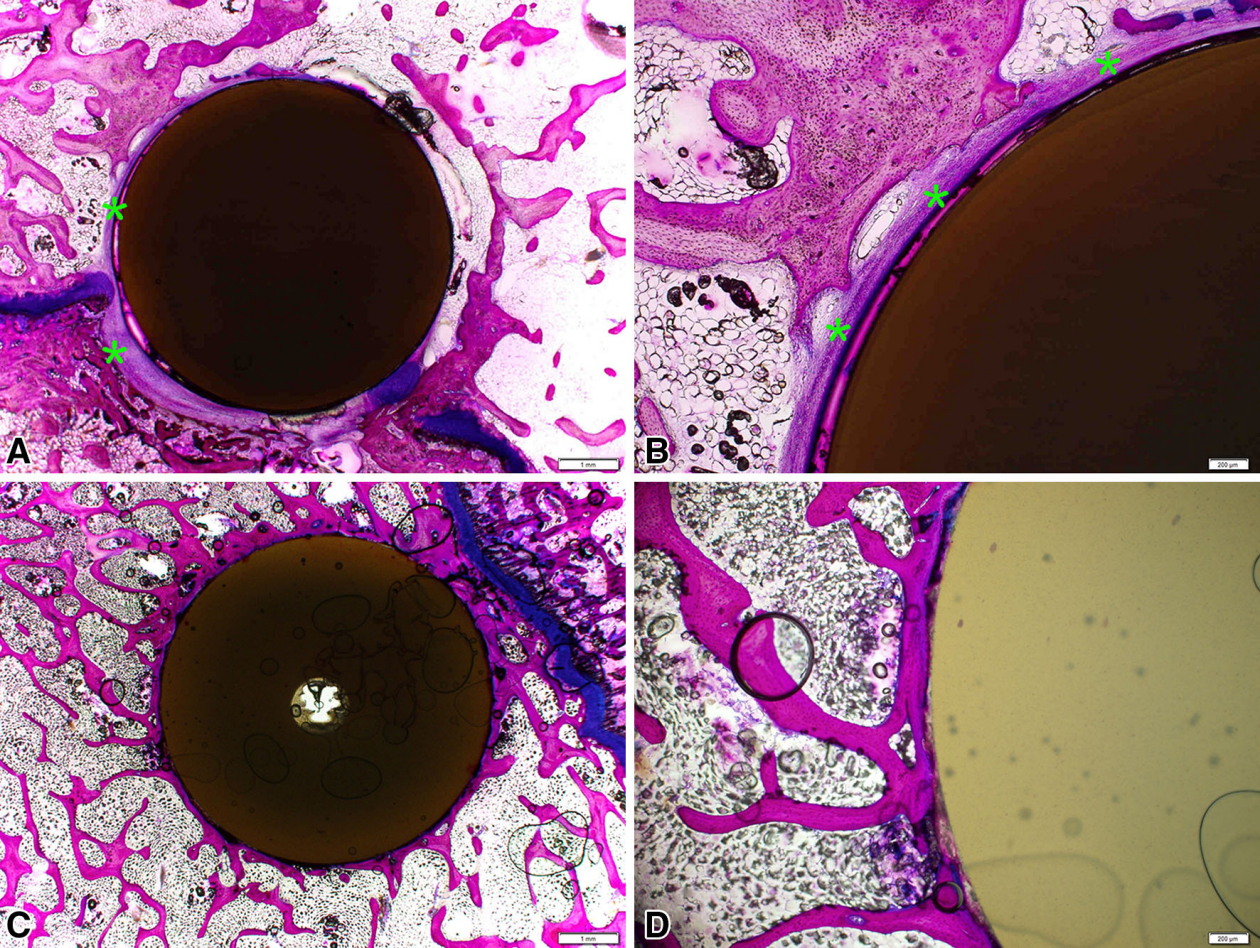
Bone ongrowth in cancellous sites for PEEK at 12 weeks (**A**, **C**) and PEEK-HA (**B**, **D**) demonstrated the presence of fibrous tissue interface for PEEK (*), whereas a direct bone-to-implant interface was observed for PEEK-HA at the magnification used. Magnification bars = 1 mm for A and C and 200 μm for **B** and **D**.

Faxitron radiographs did not reveal any adverse bony reactions in terms of resorption to either group at 4 or 12 weeks in the cortical or cancellous sites. PMMA histology revealed the typical fibrous tissue interface and the lack of any inflammatory cell types with PEEK or PEEK-HA at 4 and 12 weeks in cortical and cancellous sites (Figs. 2, 3). Areas of direct bone to implant contact were noted with the PEEK-HA dowels at 4 and 12 weeks in cortical and cancellous sites (Figs. 2, 3).

### Cervical Interbody Fusion Study in Sheep

**Fig. 4:**
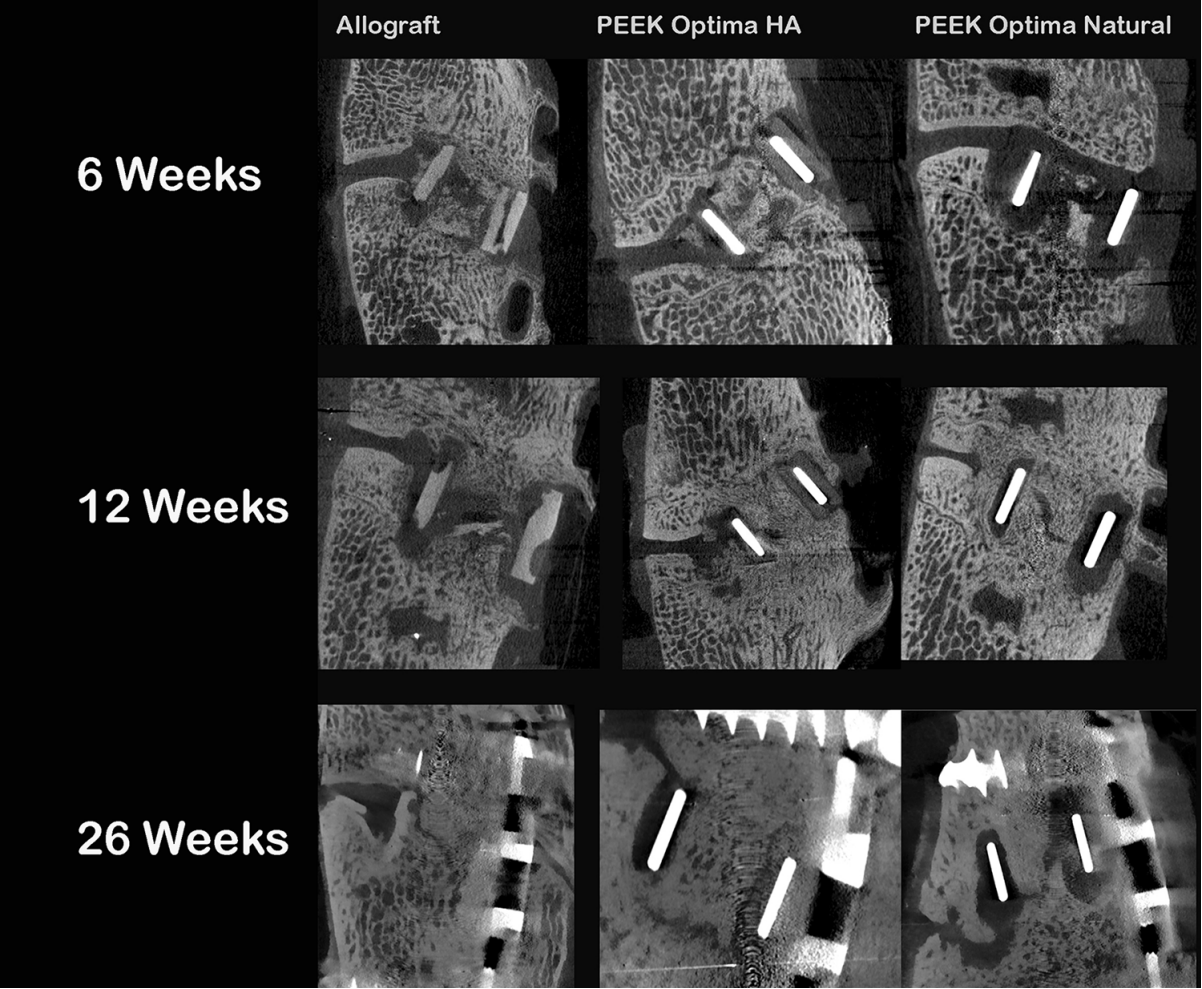
Micro-CT at 6, 12, and 26 weeks for allograft, PEEK-HA, and PEEK demonstrated progression in fusion versus time for all groups. Fracture and resorption of the allograft cages were observed. Fusions remodeled with time for all groups.

Faxitron radiographs did not reveal any adverse bony reactions, whereas resorption and remodeling of the allograft spacers were noted. The PEEK and PEEK-HA devices remained intact throughout the implantation periods. Micro-CT demonstrated fracture and resorption of the allograft interbody devices that were not detected in the Faxitron radiographs. Resorption of the allograft interbody spacers was noted at all time points. Fracture of the allograft interbody spacers was noted in three of five at 6 weeks; two of four at 12 weeks; and one of four at 26 weeks for a total six of the 13 sites (46%) during the in vivo implantation period. Micro-CT analysis demonstrated that new bone formation was greater with the PEEK-HA devices compared with PEEK at 6 weeks (Table 2). The quality of new bone bridging between the vertebral bodies and contributing toward fusion was more mature in the PEEK-HA group compared with PEEK at all time points (Fig. 4). A qualitative examination of the μCT images supported greater direct bone contact with the PEEK-HA devices compared with PEEK, and this was more evident at 6 and 12 weeks while the fusion was still maturing and remodeling.

**Table 2:** CT grading results

Parameter	Group	6 weeks	12 weeks	26 weeks
New bone	Allograft	2.6 ± 0.9	2.0 ± 0.8	1.8 ± 1.0
	PEEK Optima HA	2.0 ± 0.8	2.8 ± 0.5	3.0 ± 0.0
	PEEK Optima Natural	1.0 ± 0.0	3.0 ± 0.0	3.0 ± 0.0
Quality	Allograft	1.2 ± 1.3	0.5 ± 0.6	0.8 ± 1.5
	PEEK Optima HA	1.0 ± 1.4	1.5 ± 1.3	1.8 ± 1.0
	PEEK Optima Natural	0.0 ± 0.0	0.8 ± 1.0	1.8 ± 1.5
Contact	Allograft	1.8 ± 1.3	1.3 ± 1.5	2.0 ± 1.2
	PEEK Optima HA	0.5 ± 0.6	1.5 ± 1.0	1.3 ± 0.5
	PEEK Optima Natural	0.0 ± 0.0	0.5 ± 0.6	1.0 ± 1.4

Data are presented as mean ± SD.

**Fig. 5:**
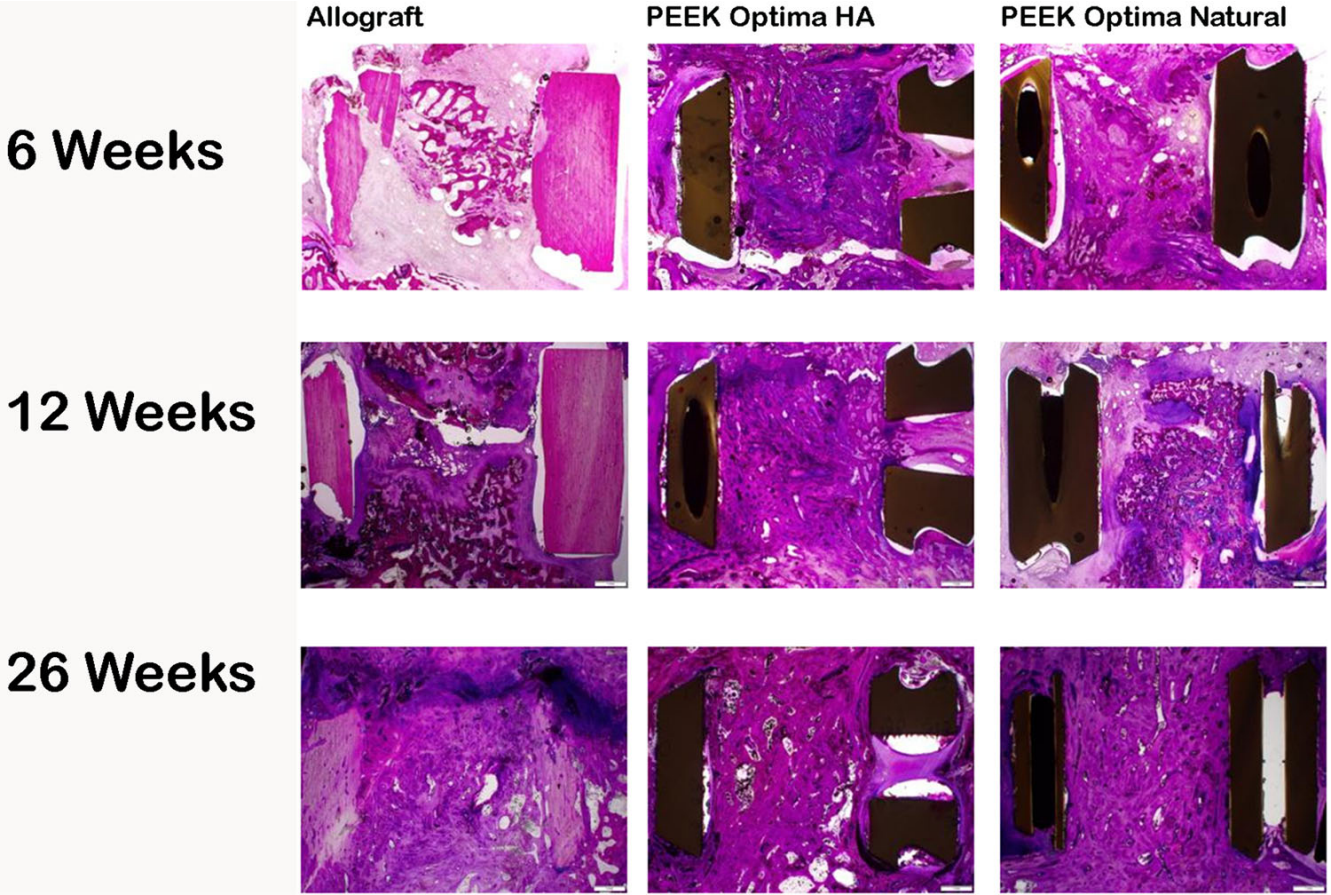
Macroscopic overview of PMMA histology at 6, 12, and 26 weeks for allograft, PEEK-HA, and PEEK Natural demonstrated a progression in fusion versus time for all groups. Allograft cages fractured as well as resorbed with time, whereas no failure was observed in the PEEK-HA or PEEK devices. A fibrous tissue interface was present for PEEK at 6 and 12 weeks. Direct bone contact was observed with PEEK-HA at 12 weeks. All fusions remodeled with time and were mature by 26 weeks.

Histology confirmed the biocompatibility of PEEK as well as PEEK-HA with no evidence of inflammatory cell types at the interface at 6, 12, and 26 weeks (Fig. 5). This was supported by the ISO 10993-6 evaluation (data not shown).

The graft inside the PEEK-HA devices qualitatively appeared more robust at 6 and 12 weeks compared with the graft inside the PEEK devices. These differences were less evident at 26 weeks but remained suggestive of a more mature graft in the PEEK-HA devices compared with PEEK. In contrast, there was substantial resorption of the allograft implants and fracture of the devices as noted in the μCT results. Allograft devices showed a high degree of new bone formation and incorporation into the surrounding bone. This was countered, however, by the high degree of resorption combined with device fracture.

## Discussion

Controlling the surface interactions at the bone-implant interface has the potential to influence the in vivo responses to a device. Modifying properties of a material either through surface coatings or topography can increase bone ingrowth or ongrowth to orthopaedic biomaterials [2–5, 24–26, 28]. Direct bone ongrowth to PEEK-HA could provide a better interface than the nonreactive fibrous layer reported for PEEK alone in bone [28]. We hypothesized that the incorporation of HA into the PEEK matrix would enhance the in vivo response at the bone–implant interface. Using in vivo sheep models, we asked: (1) Does PEEK-HA improve cortical and cancellous bone ongrowth compared with PEEK alone? (2) Does PEEK-HA improve bone ongrowth and fusion outcome in a more challenging cervical fusion model? The current studies demonstrate incorporating HA into PEEK provides a more favorable environment than PEEK alone for bone ongrowth and spinal fusion.

Although the two models used in the current study represent increasing complexity of surgery, loading, and kinematics, they remain limited for direct comparisons with human pathology. The long bone ongrowth model [2–5, 24–26, 28] is limited because the implants are contained in a closed defect and are not under direct load. The cervical fusion model is more demanding with implants under load and motion between the vertebral bodies and the device endplates. The small sample size and lack of followup beyond 26 weeks are also limiting factors. Nevertheless, preclinical animal models allow for examination of devices and interfaces using μCT and histology beyond the scope of human studies.

Our results demonstrate incorporating HA into PEEK qualitatively enhanced bone ongrowth in cortical and cancellous sites, whereas PEEK alone presented the nonreactive fibrous tissue interface as reported previously [16, 20, 28]. The fusions across all groups improved with time demonstrating an increase in new bone formation within the interbody devices. The allograft interbody device showed the most variability in new bone formation, device fracture, and fusion quality. The allograft cage was the most “biologically” active with new bone formation directly on the surfaces of the implant. Although the benefit of the HA incorporation into the PEEK was not differentiated at the clinical or radiographic levels, micro-CT and histology showed direct bone contact rather than a nonreactive fibrous tissue layer at the implant interface. The fusion histology was also more mature with the PEEK-HA group compared with PEEK alone or allograft spacers.

HA at the surface of PEEK provides an osteoconductive surface, which supports bone apposition. This material is being used clinically and future reports in humans will provide a bridge between the preclinical and clinical results. The application of new materials to improve clinical outcome and participate actively with the biologic process involved in healing represents an exciting area of new research. This coupled with increased understanding of the ideal geometry for interbody devices will continue to push this field forward.
